# Evolution of the p53-MDM2 pathway

**DOI:** 10.1186/s12862-017-1023-y

**Published:** 2017-08-03

**Authors:** Emma Åberg, Fulvio Saccoccia, Manfred Grabherr, Wai Ying Josefin Ore, Per Jemth, Greta Hultqvist

**Affiliations:** 1 Department of Medical Biochemistry and Microbiology, Uppsala University, BMC Box 582, SE-75123 Uppsala, Sweden; 2 Department of Pharmaceutical Biosciences, Uppsala University, BMC, Box 591, SE-75124, Uppsala, Sweden

**Keywords:** p53, MDM, Co-evolution, Phylogeny

## Abstract

**Background:**

The p53 signalling pathway, which controls cell fate, has been extensively studied due to its prominent role in tumor development. The pathway includes the tumor supressor protein p53, its vertebrate paralogs p63 and p73, and their negative regulators MDM2 and MDM4. The p53/p63/p73-MDM system is ancient and can be traced in all extant animal phyla. Despite this, correct phylogenetic trees including both vertebrate and invertebrate species of the p53/p63/p73 and MDM families have not been published.

**Results:**

Here, we have examined the evolution of the p53/p63/p73 protein family with particular focus on the p53/p63/p73 transactivation domain (TAD) and its co-evolution with the p53/p63/p73-binding domain (p53/p63/p73BD) of MDM2. We found that the TAD and p53/p63/p73BD share a strong evolutionary connection. If one of the domains of the protein is lost in a phylum, then it seems very likely to be followed by loss of function by the other domain as well, and due to the loss of function it is likely to eventually disappear. By focusing our phylogenetic analysis to p53/p63/p73 and MDM proteins from phyla that retain the interaction domains TAD and p53/p63/p73BD, we built phylogenetic trees of p53/p63/p73 and MDM based on both vertebrate and invertebrate species. The trees follow species evolution and contain a total number of 183 and 98 species for p53/p63/p73 and MDM, respectively. We also demonstrate that the p53/p63/p73 and MDM families result from whole genome duplications.

**Conclusions:**

The signaling pathway of the TAD and p53/p63/p73BD in p53/p63/p73 and MDM, respectively, dates back to early metazoan time and has since then tightly co-evolved, or disappeared in distinct lineages.

**Electronic supplementary material:**

The online version of this article (doi:10.1186/s12862-017-1023-y) contains supplementary material, which is available to authorized users.

## Background

Cancer has been observed in virtually all vertebrates, regardless of body size and lifespan, while cancer-like growths have been reported in protostome invertebrates [1]. In mammals, such as humans and mice, there are protective systems in place. As part of this system, p53, often referred to as the “guardian of the genome”, plays the important role as an anti-cancer protein. p53 is a transcription factor responsible for regulating the fate of the cell, for example during stress and DNA damage [2]. MDM2 is the primary negative regulator of p53, keeping p53 at appropriate levels by ubiquitination in normal functioning cells [3]. Upon stress, p53 is activated and fulfils its role as a tumor suppressor protein, for example by inducing apoptosis. p53, or the p53 pathway, is disabled in roughly half of all human cancers [4]. Consequently, the prominent role of p53 and MDM2 in tumor suppression makes them outstanding targets for drug design [5], as well as highly interesting for detailed evolutionary studies [6, 7].

p53 shares ancestry with two other transcription factors, p63 and p73, which are paralogs of p53 [8]: p63 is responsible for skin and epithelial development, while p73 plays a role in neuronal development and differentiation [9]. In vertebrates, MDM2 belongs to a family with two members, MDM2 and MDM4. To date, members of the p53/p63/p73 and MDM families have been reported in chordates, but also in non-chordate species, such as *Mytilus trossulus* (bay mussel) [10], *Ixodes scapularis* (deer tick) [11] and *Trichoplax adhaerens*, a small (<1 mm) animal that is the only known living representative of the phylum *Placozoa* [12]. Thus, the ubiquitous presence of both proteins suggests that they were present in the common ancestor of all present-day animals, and we thus refer to these proteins as p53/p63/p73^ancestor^ and MDM^ancestor^, respectively.

Interestingly, the evolutionary history of the p53/p63/p73 family has proven difficult to fully understand, and there is no published phylogenetic tree that agrees with the generally accepted tree of life for animal evolution [13–15]. For the MDM family no comprehensive phylogenetic tree has been published. To investigate the interaction between p53/p63/p73 and MDM, we have re-examined their evolutionary history. We found a strong correlation in the conservation of the interacting domains, p53/p63/p73 TAD and MDM p53/p63/p73BD. Loss of one of the domains is associated with the lack of the other domain, with few exceptions, demonstrating their functional dependence. By utilizing conserved amino acid sequences in domains with retained function, we could infer a phylogenetic relationship of metazoan genes containing p53/p63/p73 TAD and p53/p63/p73BD, respectively. These trees include both vertebrate and invertebrate species, and are consistent with the species evolution for both p53/p63/p73 and MDM. Finally, we have examined the evolution of the p53/p63/p73 TAD domain on a molecular level with regard to protein disorder and regulatory properties. We observed similarities in the phosphorylation pattern of vertebrate p53 and mollusk and annelid p53/p63/p73, which imply that the functional properties of regulation through phosphorylation were present already in the ancestor of deuterostomes (e.g. Chordata) and protostomes (e.g. Mollusca and Arthropoda).

## Results

### Emergence and loss of domains within the p53/p63/p73 family

**Fig. 1:**
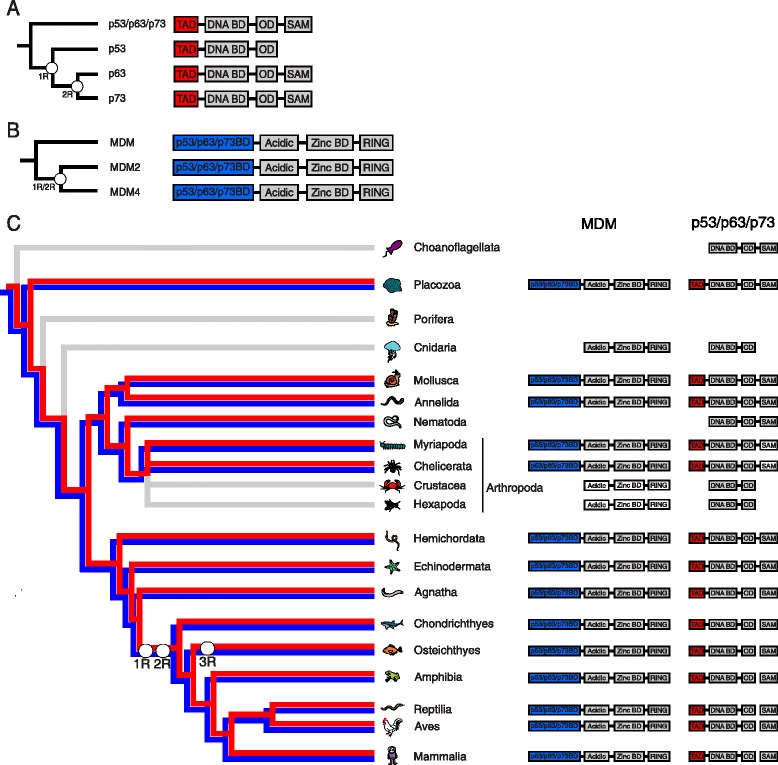
Domain organization of (**a**) the p53/p63/p73 protein family comprising the transactivation domain (TAD), DNA binding domain (DNA BD), oligomerisation domain (OD) and the sterile alpha-motif (SAM) domain. **b** the MDM protein family containing the p53/p63/p73-binding domain (p53/p63/p73BD), the Acidic domain, a zinc binding domain (Zinc BD) and a RING domain. **c** Species tree displaying the existence of p53/p63/p73 TAD (in *red*) and MDM p53/p63/p73BD (in *blue*) along with the presence of the other domains in the respective protein. *Grey branches* in the tree illustrate that p53/p63/p73BD and TAD is not present. The domains displayed in *white* indicate that the domains are present in a few organisms in that specific lineage, but in the majority of the examined species the domain could not be found. The SAM domain was lost in p53 after the whole genome duplication, denoted 1R in the tree, but is retained in vertebrate p63 and p73. This variability is illustrated with absence of lines connecting the OD and SAM domain. The second whole genome duplication is denoted 2R

Four distinct domains: the transactivation domain (TAD), the DNA binding domain (DNA BD), the oligomerisation domain (OD) and the sterile alpha motif (SAM) (Fig. 1a) are common in proteins from the p53/p63/p73 family. By extensive BLAST searches in metazoan genome databases, we found 342 unique p53/p63/p73 family genes belonging to 183 species. We could confirm the presence of two p53/p63/p73-like genes in the unicellular choanoflagellate *Monosiga brevicollis* [16]. The two *Monosiga brevicollis* p53/p63/p73 genes do not contain the TAD but only the DNA BD and the OD, whereas the SAM domain is present in one of the genes but is missing in the other. As compared to vertebrates, the most distantly related p53/p63/p73 gene comprising TAD is that of *Trichoplax adhaerens* (a multicellular eukaryote, the only member of the phylum Placozoa) [12] (Fig. 1c). Partial or complete gene loss has resulted in complete lack of p53/p63/p73 in Porifera (sponges), and in a truncated version of p53/p63/p73 in Cnidarian species (including e.g., corals and jellyfish), in which the TAD and SAM domains have been lost. The loss of TAD and SAM appears to be a restricted event in these branches since the domains can be identified in sister groups (Fig. 1c). The gene is present in both deuterostome and protostome species suggesting that it appeared early in metazoan (animal) evolution and was present in the common ancestor of animals [17].

Protostomes can be divided into four phyla, where closer ancestry is shared between Annelida (ringed worms) and Mollusca, and between Arthropoda and Nematoda (roundworms), respectively. In species from Annelida and Mollusca, all four p53/p63/p73 domains are conserved, but within the Arthropoda phylum, certain domains have been lost (Fig. 1c). Species in the Arthropoda subphyla Chelicerata (including e.g., scorpions and spiders) and Myriapoda (e.g., millipeds) have a p53/p63/p73 gene that contains all four domains while species from subphyla Hexapoda (e.g., insects), and Crustacea (e.g., crayfish and crabs) contain a truncated p53/p63/p73 gene with the DNA BD and OD. Similarly, in p53/p63/p73 from Nematoda, the TAD and SAM domains have been lost, and only the DNA BD and OD are present (Fig. 1c). All extant phyla of deuterostomes (including Chordata, Hemichordata and Echinodermata) have p53/p63/p73 genes comprising all four domains, which implies that the ancestor of deuterostome species also contained a p53/p63/p73 gene with all domains. Following two whole genome duplications early in the vertebrate lineage [18], the three paralogs p53, p63, and p73 emerged. p63 and p73 have retained all four domains, while the SAM domain was lost in the p53 lineage and replaced with a C-terminal disordered domain involved in protein-protein interactions [19].

### Duplications within the p53/p63/p73 family

There are several papers that have analyzed the number of p53/p63/p73 genes and which domains these contain in different species in order to understand the p53/p63/p73 evolution [6, 20, 21]. These papers often refer to the genes with the SAM domain in invertebrates as p63/p73 or p63/p73-like and to the ones lacking the SAM domain to p53 or p53-like. To infer such a relationship is however not straightforward since domains are frequently lost during evolution and hence lack of a particular domain in a protein does not confirm close relationship with another protein lacking the same domain. The SAM domain has indeed been lost at multiple occasions during the evolution of the p53/p63/p73 family. A recent study by dos Santos et al. where they published a phylogenetic tree and included duplicates of invertebrates shows that there has been multiple duplications in the evolution of the p53/p63/p73 family [13]. For instance, the choanoflagellate *Monosiga brevicollis* have one copy of p53/p63/p73 with the SAM domain and one without and these are more similar to each other than to the vertebrate p53, p63 and p73 genes according to the results in dos Santos et al. Furthermore, in several hexapod species in the arthropod lineage the p53/p63/p73 gene has been duplicated at different time points: *Aedes aegypti, Anopheles gambiae* and *Culex quinquefasciatus* p53/p63/p73 gene seem to have been duplicated in the ancestor of these species as they cluster together while the p53/p63/p73 gene in *Nasonia vitripennis* and *Tribolium castaneum* have been duplicated in two separate events. In another genus of non-vertebrate chordates, *Branchiostoma floridae*, one of the p53/p63/p73 genes variants has lost the SAM domain while the other has retained it, they do not cluster in the phylogenetic tree, however they are neither located in a way that implies closer relationship to any of the vertebrate p53, p63 or p73 [13]. A more recent duplication of the p53/p63/p73 gene can be found in the chordate but non-vertebrate tunicate *Ciona intestinalis*. In conclusion, the p53/p63/p73 genes have been duplicated multiple times during the evolution. Furthermore, after the whole genome duplications in the vertebrate lineage leading to fishes, reptiles and mammals the three distinct p53, p63 and p73 genes were retained in the majority of species. However, gene duplications in vertebrates can also be observed, for example, there are 20 copies of p53 in the African elephant *Loxodonta africana* [22].

### Emergence and loss of domains within the MDM family

Similarly to p53/p63/p73, we performed BLAST searches in metazoan genome databases for MDM, and found 166 unique MDM family genes belonging to 98 species. The MDM protein family consists of four domains, the p53/p63/p73-binding domain (p53/p63/p73BD), the Acidic domain, the zinc binding domain (Zinc BD), and the RING domain (Fig. 1b). An MDM protein comprising all four domains was previously identified in the multicellular Placozoan, *Trichoplax adhaerens* [12]. The MDM gene is not present in Porifera (sponges), but it can be found within the Cnidaria phylum. However, Cnidaria MDM (i.e., from the species *Nematostella vectensis*, *Hydra vulgaris* and *Acropora digitifera*) lacks the p53/p63/p73BD (Fig. 1c). Since the MDM gene is present in deuterostomes and protostomes, it was consequently present in the common ancestor of extant multicellular animal species. Certain domains of MDM have been lost in the protostome lineage similarly to what we observe for p53/p63/p73 (Fig. 1c). In the Mollusca, Annelida and Arthropoda subphyla Myriapoda and Chelicerata, an MDM gene comprising all four domains was identified. However, in Nematoda, the whole gene has disappeared. In the Arthropoda subphyla Hexapoda and Crustacea, the acidic domain, zinc binding domain and the RING finger domain can be identified in a few species, but not the p53/p63/p73BD. In deuterostome species, all four domains are present in both paralogs, MDM2 and MDM4.

### Loss of the TAD domain in p53/p63/p73 correlates with the loss of the p53/p63/p73BD in MDM

The interaction between p53 TAD and MDM2 p53/p63/p73BD is important in mammals, since it is involved in tumor suppression. The origin of the interaction between the domains dates back to the time of early metazoan species [12]. Similar to p53/p63/p73, the MDM gene is not present in Porifera (sponges), but can be found within the Cnidaria phylum. However, the interaction domains in MDM and p53/p63/p73 in Cnidaria are both missing (Fig. 1c). A similar correlation between loss of p53/p63/p73BD in MDM and loss of TAD in p53/p63/p73 was observed in protostomes. For example, species belonging to the Mollusca and Annelida phyla and the Arthropoda subphyla Chelicerata and Myriapoda all contain four p53/p63/p73 domains, as well as the p53/p63/p73BD of MDM. Interestingly, the p53/p63/p73BD in MDM in the Arthropoda subphyla Chelicerata and Myriapoda species *Stegodyphus mimosarum* (african social velvet spider), *Ixodes ricius* (castor bean tick), *Ixodes scapularis* (deer tick), *Metaseiulus occidentalis* (western predatory mite) and the *Strigamia maritima* (centipede), is less conserved in length compared to the p53/p63/p73BD in vertebrate, annelid and mollusk species. Likewise, the p53/p63/p73 TAD from these species contains a less conserved MDM binding motif. On the other hand, in the Arthropoda subphyla Hexapoda and Crustacea, we could only find truncated versions of p53/p63/p73 and MDM where the interaction domains is not present. Likewise, all species in the Nematoda phylum lack the whole MDM protein and p53/p63/p73 TAD. By contrast, all deuterostome species contain all MDM domains, as well as the p53/p63/p73 TAD. Thus, we find a clear correlation between presence of p53/p63/p73 TAD and the p53/p63/p73BD in MDM. This suggests a strong and ancient, yet dynamic co-evolution of the interaction domains TAD and p53/p63/p73BD in the p53/p63/p73-MDM regulatory pathway. However, there are a few cases that are not clear, which are detailed below.

### Species that might not conform to the co-evolution hypothesis

**Fig. 2:**
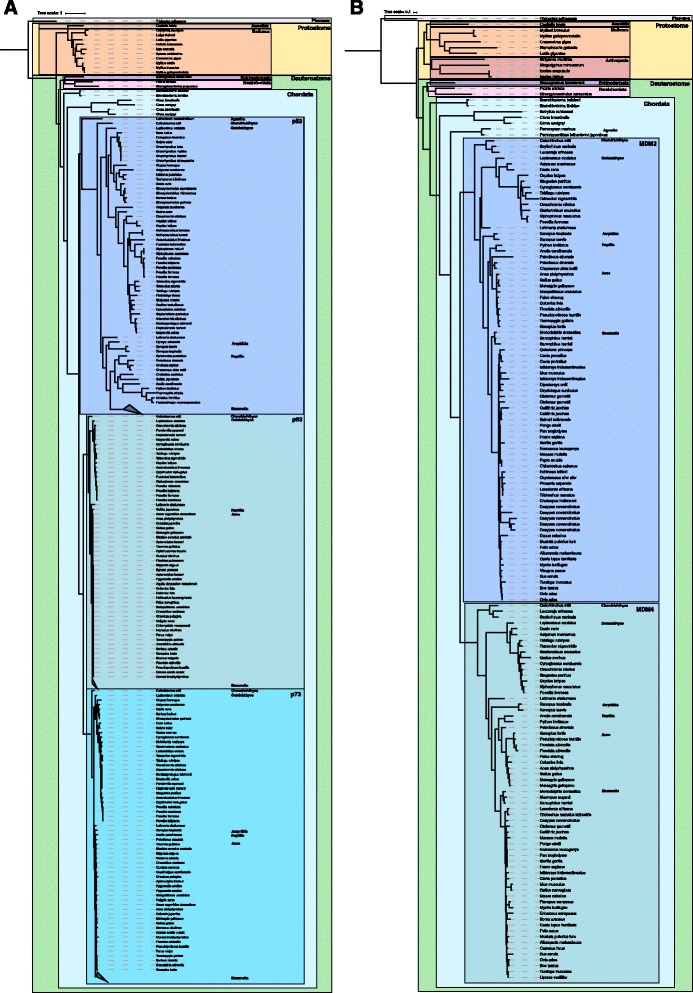
**a** Phylogenetic tree based on multiple sequence alignment of the p53/p63/p73 protein family only including species with the TAD. The evolutionary relations are the same as what is generally accepted regarding species evolution and whole genome duplications. The Placozoa sequence is most distantly related to all the other genes in the tree and was therefore used as an outgroup. **b** Phylogenetic tree based on multiple sequence alignment of the MDM protein family only including species with the p53/p63/p73BD. The evolutionary relations are the same as what is generally accepted regarding species evolution and whole genome duplications. The Placozoa sequence is most distantly related to all the other genes in the tree and was therefore used as an outgroup

While the co-evolution of p53/p63/p73 and MDM appears strong, some of our data are inconclusive. Among invertebrates, we found species in the Mollusca phylum having p53/p63/p73 with the TAD but not MDM, for example *Haliotis tuberculat* (a sea snail), *Euprymna scolopes* (bobtail squid), *Spisula solidissima* (Atlantic sea clam) and *Loligo forbesii* (long-finned squid). By contrast, in *Biomphalaria glabrata* (ram’s horn snail), an MDM with a p53/p63/p73BD was found, while its p53/p63/p73 lack the TAD. However, since all these genomes have relatively poor sequence coverage, and since there are related species, for example *Mytilus trossulus* (bay mussel), *Crassostrea gigas* (Pacific oyster) and *Lottia gigantea* (owl limpet), where both interaction domains are present, it is likely that all Mollusca species contain the gene with the interaction domain (Fig. 2a, b). In the majority of deuterostome species, the same paralogs are present: in the p53/p63/p73 family, the three distinct proteins p53, p63 and p73 and in the MDM family, the two proteins MDM2 and MDM4. Species belonging to the Chondrichthyes phylum (cartilaginous fish), such as *Scyliorhinus canicula* (small-spotted catshark) and *Leucoraja erinacea* (little skate) appear to not have a p53, p63 or p73 protein, but contain MDM2 and MDM4. On the other hand, *Callorhinchus milli* (Australian ghostshark), which also belongs to the Chondrichthyes phylum, contains p53, p63, p73, MDM2 and MDM4 (including the p53/p63/p73BD), which leads us to believe that the missing sequences among Chondrichthyes might be due to poor sequencing coverage. In Osteichthyes (bony fish), Reptilia, and Mammalia, there are certain species in which we cannot identify all p53, p63, p73, MDM2 and MDM4 and/or their respective interaction domain; however, the majority of the species in a phylum contains the genes. We also further investigated the previous notion that p53 is missing from the genome assemblies in the majority of species in the phylum Aves (birds) [13]. While not present in any avian genome assembly, p53 mRNA has been found in the published transcriptomes of two birds, *Gallus gallus* (Chicken) and *Pseudopodoces humilis* (ground tit). The *Gallus gallus* p53 gene has all four-domains, whereas the *Pseudopodoces humilis* p53 gene only contains the DNA-BD and OD. The high GC content of about 65% indicates that p53 is located in one of the GC rich microchromosomes, which are difficult to assemble due to sequencing bias and low complexity. Fragments of the p53 mRNA could also be found in the transcriptomes of two other bird species from different clades, *Columba livia* (pigeon) and *Erythrura gouldiae* (gouldian finch, personal communication with Malgorzata Anna Gazda), suggesting that p53 is present in all bird species, albeit difficult to detect due to its high GC content.

### Phylogeny of proteins containing the interacting domains produces phylogenetic trees that follow the species evolution

**Fig. 3:**
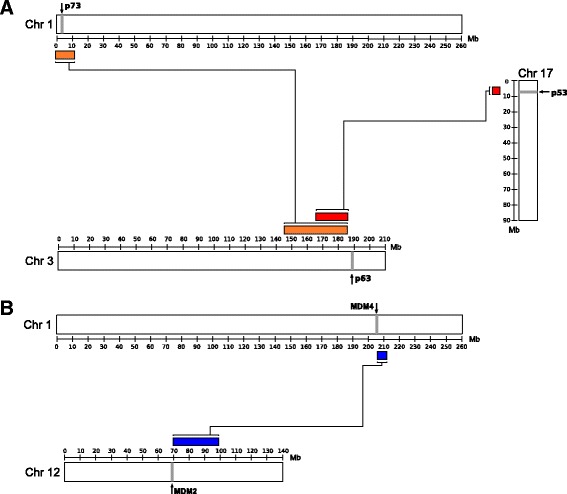
Paralogous blocks descended from the two whole genome duplication events that happened prior to the emergence of bony vertebrates. The localization of the genes is illustrated with a *grey line* and the paralogons have the same color. **a** A region on an ancestral chromosome was duplicated and can in humans be found in chromosome 3, 1 and 17 in which p63, p73 and p53 are localized, respectively. **b** A region on an ancestral chromosome was duplicated and can in humans be found in chromosome 1 and 12 where MDM2 and MDM4 are localized, respectively [18]

There have been several attempts to solve the evolutionary history of the p53/p63/p73 protein family [6, 13–15, 20], but so far no phylogenetic tree, including both vertebrate and invertebrate species, has been published that agrees with the evolution of species. The phylogeny of MDM has been sparsely investigated, and the best published tree comprises only five vertebrates and three invertebrates species [23]. Due to less structural constraints, intrinsically disordered regions, like the p53/p63/p73 TAD, are allowed to substitute at a faster rate compared to structured regions [24, 25]. Since we observe a strong co-evolution of the two interacting domains, p53/p63/p73 TAD and MDM p53/p63/p73BD, the species that contain these two domains are very likely to have retained their interaction and function limiting the amino acid substitutions and improving the likelihood of a correct alignment. We were therefore curious to examine the phylogeny of p53/p63/p73 and MDM only including species having the interaction domain to investigate the phylogenetic relationship. Thus, we reconstructed a phylogenetic tree of the p53/p63/p73 family only including species containing the TAD (Fig. 3a) and a tree of the MDM family only including species containing the p53/p63/p73BD (Fig. 3b). Our analysis includes 111 and 84 vertebrate and 15 and 14 invertebrate species for p53/p63/p73 and MDM, respectively, resulting in phylogenetic trees that follow the evolution of species almost perfectly, according to interactive Tree Of Life [26].

### Co-localization of genes on paralogons confirms that p53/p63/p73 and MDM2/MDM4 result from whole genome duplications

In local gene duplications, the two duplicated genes are located in the proximity of each other, while after whole genome duplications, the duplicated gene is found on a paralogous block resulting from recombination of chromosomes. The existence of paralogons has been confirmed by comparing the chromosomal location of duplicated human genes with the location of the evolutionary connected genes in invertebrate species as *Drosophila melanogaster* and *Caenorhabditis elegans*, which did not undergo whole genome duplications [27]. The duplicated genes were further investigated by phylogenetic and molecular clock analysis to find the time point of the duplication, which was estimated to be around the time of early vertebrate evolution [18]. Present day mammalian p53, p63 and p73, as well as MDM2 and MDM4, have been suggested to result from these two whole genome duplications in the vertebrate lineage, only due to their time point of divergence [13, 28]. That the duplications occur at the time point of the whole genome duplications is supported by our phylogenetic analysis, where the time of duplication happened after the divergence of Vertebrata and Agnatha (Jawless fish). For the p53/p63/p73 family, one copy was subsequently lost, and in case of MDM, two copies were lost. To confirm that the p53/p63/p73 and MDM family genes evolved from whole genome duplication events, we analyzed genes that are co-localized in paralogous chromosomal regions (synteny). p63 is located on chromosome 3, p53 is located on chromosome 17, and p73 is located on chromosome 1. These three regions form a paralogous block [18], hence supporting that the vertebrate p53/p63/p73 family members arose through whole genome duplications (Fig. 3a). Likewise, the location of MDM2 and MDM4 can be traced to a paralogous block in chromosome 12 and 1, respectively (Fig. 3b). These results strongly suggest that the p53/p63/p73 and MDM genes arose from the whole genome duplications in the vertebrate lineage. In teleost fish, an additional whole genome duplication occurred after the divergence from present day tetrapods [29], implying that two copies of p53, p63, p73, MDM2 and MDM4, respectively, can be present in some teleost fish species. However, we did not find any instances where the duplicated genes were preserved suggesting they have been lost, which is a common event.

### Evolution of phosphorylation sites in the p53-TAD domain

**Fig. 4:**
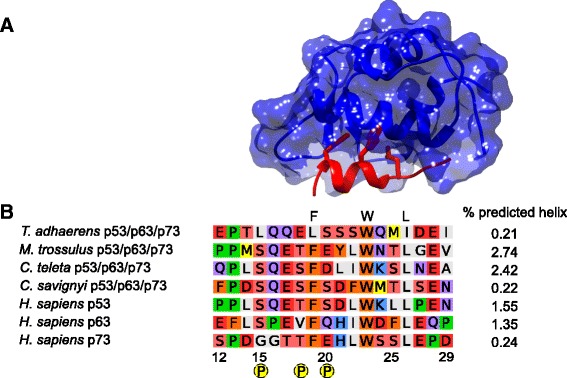
**a** Crystal structure of the complex between mouse p53 TAD (*red*) and the p53/p63/p73BD of MDM2 (*blue*) (PDB entry: 1YCR) [37]. The residues in p53 TAD shown as sticks are the three conserved residues in the FxxxWxxL motif. **b** Alignment of the TAD of selected species. Amino acid numbering and phosphorylation sites are according to human p53. Agadir prediction [39] of the helical propensity in percent is shown beside the alignment for the different species. The color-coding is according to eBioX alignment tool

Studies on mammalian p53 TAD have shown that it is intrinsically disordered in the free state, but adopts a helical structure when binding to MDM2 and other interaction partners (Fig. 4a) [30]. Posttranslational modifications help to regulate the function and affinities for different binding partners, and are common in regions with intrinsic disorder [31]. Human p53 TAD has three possible phosphorylation sites, at Ser15, Thr18 and Ser20 (Fig. 4b). Especially, the phosphorylation of Thr18 in p53 TAD increases the affinity for proteins activating p53, such as CBP [32] and p300 [33]. The affinity is increased in an additive manner for each site that becomes phosphorylated [33]. On the other hand, phosphorylation of Thr18 decreases the affinity for MDM2 [30]. We were interested to see when this phosphorylation pattern appeared, and if it is conserved in evolution. Our result shows that all three putative phosphorylation sites are conserved in the p53 vertebrate linage. However, only Ser15 is conserved in the p63 lineage. Among p73 vertebrates Thr18 is instead conserved, and additional Ser and Thr residues have emerged, but are not confirmed phosphorylation sites according to the PhosphositePlus webpage [34]. The vertebrate p53 phosphorylation sites Ser15 and Thr18 are present in mollusk species, whereas in *Capitella teleta* (a polychaete worm from the phylum Annelida), only Ser15 is conserved and has a Ser residue at position 18 instead, which is also a putative phosphorylation site. In Chordata species that did not undergo whole genome duplication, such as *Ciona intestinalis* and *Ciona savignyi*, the phosphorylation sites in p53/p63/p73 TAD are Ser15, Thr18 and Ser20, while the echinoderm species *Patiria miniata* and *Strongylocentrotus purpuratus* contain the putative phosphorylation sites Ser15 and Thr18. Thus, the mollusk and annelid p53/p63/p73 phosphorylation pattern is more similar to the pattern in echinoderm p53/p63/p73 and vertebrate p53 compared to vertebrate p63 and p73, suggesting that the present day vertebrate p53 pattern (and thus possibly the regulation through phosphorylation) was present already in the deuterostome/protostome ancestor (Fig. 4b).

### Evolution of residual helicity in p53/p63/p73 TAD domain

The molecular evolution of intrinsically disordered proteins (IDPs) is known to have less constraints and is more prone to insertions and deletions compared to structured domains [35]. However, binding motifs, amino acid composition, and the length of IDPs are generally conserved [25]. Computational analysis of the primary structure in disordered regions in different species can provide some insights with regard to important residues that have persisted in the evolutionary process. Prolines are of interest when considering the residual helicity, since they sterically hinder the continuation of helical structures. The human p53 TAD has two N-terminal prolines and one C-terminal proline present at the respective end of the FxxxWxxL binding motif (Fig. 4). In a recent study [36] the prolines of human p53 TAD were mutated to alanine to assess the effect of the helical structure on binding affinity to MDM2 p53/p63/73BD and general function of p53. The study revealed that the N-terminal prolines (position 12 and 13) have no effect on binding, while mutation of the C-terminal proline (position 27) results in higher residual helicity and a higher affinity for the p53/p63/73BD. The C-terminal proline is conserved in the vertebrate lineage for p53. Human p63 and p73 also have a proline C-terminal of the TAD binding motif, at position 65 and 24, respectively, while invertebrate p53/p63/p73 TAD lacks a proline in this position (Fig. 4b). Published structures of p53 TAD [37] (Fig. 4a) and p73 TAD [38] in complex with MDM2 indicate a helical structure between positions 18-26 and 14-21, respectively. Agadir predictions [39] of the helical content of TAD from human p53, p63 and p73, as well as for invertebrate p53/p63/p73, indicate a very low helical content, suggesting that the degree of disorder in the free state is preserved in evolution irrespective of the proline (Fig. 4b). However, the conserved C-terminal proline in the vertebrate lineage of p53, p63 and p73 could provide a means for TAD to modulate helicity upon binding and thus the affinity of the interaction [36].

## Discussion

Explaining the evolution of p53/p63/p73 is challenging since no phylogenetic tree including both vertebrate and invertebrate species, which follows the species evolution, has been published [13]. Phylogenetic trees including deuterostomes [15, 40] present a satisfying evolutionary relationship, while three trees including invertebrates [13, 14, 20] are not consistent with the species evolution. The relationships of species in these three trees are similarly inferred, where deuterostomes, mollusks, and annelids cluster together, while nematodes and arthropods are grouped together in another cluster. This is not in concordance with the evolution of the species, where mollusks, annelids, nematodes and arthropods should cluster together (Fig. 1c), indicating constraints in the gene family. For MDM, a comprehensive phylogenetic study has not been published, however, there are studies involving an evolutionary perspective of the protein family [7, 28]. Here, we present comprehensive phylogenetic trees for both the p53/p63/p73 and MDM family, in which the topology follows the species phylogeny and the whole genome duplications (Fig. 2a, b). We manage to do this by excluding genes that lack the two interacting domains, p53/p63/p73 TAD and MDM p53/p63/p73BD, or essential motifs in these domains.

Our phylogenetic trees of p53/p63/p73 and MDM exclude species belonging to the phylum Nematoda and the Arthropoda subphyla Hexapoda and Crustacea, since these genes lack the complete interaction domains. We believe that this particular limitation of genes is essential for the correct phylogenetic relationship since the species included have an evolutionary conserved p53/p63/p73 TAD: MDM p53/p63/p73BD interaction and hence have more similar constraints. There have been other attempts to create trees of the p53/p63/p73 family with only selected domains in the alignment. For instance dos Santos et al. made an alignment containing only the p53 DNA BD, which is conserved in all p53/p63/p73 family members, but the resulting tree did not follow the species evolution [13]. The TAD domain is intrinsically disordered and has accumulated distinct mutations in different lineages, hence contains valuable evolutionary information. Intrinsically disordered domains can be difficult to align due to the high substitution rates but the conserved FxxxWxxL motif aids in aligning the less conserved regions of the TAD domain. While the TAD domain is only a small part of the whole p53/p63/p73 gene, it is likely that the combination of the TAD and the very conserved folded domains of p53/p63/p73 provides enough information for a correct phylogenetic reconstruction. In the cases where p53/p63/p73 TAD has lost its functional connection to MDM, the substitution rate increased, resulting in sequences that could easily distort a phylogenetic reconstruction.

The human p53/p63/p73BD in MDM2 and MDM4 can both interact with TAD in p53, p63, and p73, respectively [41]. This, together with the interaction between p53/p63/p73 and MDM in bay mussel [10] implies that the interaction was present in the ancestor of deuterostomes and protostomes. The function of invertebrate p53/p63/p73 (and of p53/p63/p73^ancestor^) is thought to be protection of the germ line from DNA damage in response to stress [6], which is similar to the function of vertebrate p53. There is also evidence of leukemic-like disease in mollusks where p53/p63/p73 is up regulated [10] suggesting that p53/p63/p73 and MDM are involved in cancer in invertebrates as well as in vertebrates. Our data suggests that the TAD domain in mollusk and annelid p53/p63/p73 has a more similar phosphorylation pattern to vertebrate p53 and echinoderm p53/p63/p73 than to the vertebrate p63 and p73 family members. This leads us to propose that at the time of the split of deuterostomes and protostomes, the p53/p63/p73-MDM interaction had p53-like functionality, which has been retained in mollusk and annelid species and in p53 vertebrates. It has been suggested [6] that the ancestral and invertebrate function of p53/p63/p73 mainly resembles the p63 vertebrate function based on the presence of the conserved SAM domain and a greater sequence similarity between vertebrate p63 and invertebrate p53/p63/p73 [14]. Therefore, we also propose that some functions of p53/p63/p73^ancestor^ are more similar to that of p63 (i.e. the SAM domain functions) and others more similar to p53 (TAD domain functions). It is also possible that other functions not yet analyzed are more similar to p73, since all three family members are equally evolutionarily close to the p53/p63/p73^ancestor^.

Including all genes that have sequence similarity to MDM in the phylogenetic analysis does not produce a correct relationship according to the species tree. However, similarly to p53/p63/p73, when only species that contain the p53/p63/p73BD are included, the tree is in accordance with the whole genome duplications and species evolution. The MDM family shows highest conservation in the RING domain. The functional role of the RING domain in MDM2, which is conserved in all vertebrate species and jawless fish, is to form heterodimers with MDM4 stimulating MDM2 to ubiquitinate p53 [40]. It has been reported [42] that MDM4 has no E3-ligase activity, which raises the question whether invertebrate MDM and MDM^ancestor^ possess E3-ligase activity.

## Conclusions

In conclusion, the signaling pathway of the TAD and p53/p63/p73BD in p53/p63/p73 and MDM, respectively, dates back to the beginning of multicellular life and has since then tightly co-evolved. We have here, by only including genes containing the interaction domains for the first time constructed phylogenetic trees of both p53/p63/p73 and MDM, displaying a relationship in accordance with the whole genome duplications and species evolution.

## Methods

### Identification of p53/p63/p73 genes

p53/p63/p73 was identified in Ensembl using TBLASTN [43] (www.ensembl.org) and its gene tree (ENSGT00390000015092) was downloaded. In Uniprot (www.uniprot.org) the human p53 sequence was used as query to blast against all metazoan species, all hits were collected. The same search was performed in Ortho DB [44] (http://orthodb.org/) where all the hits were collected. Additional searches were made in NCBI (www.ncbi.nlm.nih.gov) and at the Reptilian transcriptomes webpage (http://www.reptilian-transcriptomes.org). All retrieved sequences were pooled together and duplicates were removed by using the online programme ElimDupes (www.hiv.lanl.gov/content/sequence/ELIMDUPES/elimdupes.html). The p53 TAD has a well-conserved FxxxWxxL binding motif and previous studies have shown that these are the most critical amino acids for the interaction with MDM2 [37, 45]. In the alignment we kept all sequences containing the TAD and a binding motif resembling the FxxxWxxL in amino acid character. The alignment resulted in 342 sequences from 183 species (Additional file 1: Table S1).

### Alignment and phylogenetic tree of p53/p63/p73

The amino acid alignment was done in Guidance [46] (http://guidance.tau.ac.il) using the MAFFT algorithm with the advanced option max-iterate set to 1000 and pairwise alignment option set to localpair. Gaps where removed with a gap tolerance of 95% with Gap Strip/Squeeze v2.1.0 (http://www.hiv.lanl.gov/content/sequence/GAPSTREEZE/gap.html) and this alignment was used to generate the phylogenetic tree. Alignment of the TAD is presented in Additional file 2: Figure S1 and Sequence Logos of this alignment is presented in Additional file 3: Figure S2. The best-fit model, according to Bayesian information criterion [47] (BIC) was calculated using MEGA 6 [48] and resulted in the Jones-Taylor-Thornton substitution model (JTT) model with gamma-shaped function (G) (4 categories, fixed alpha to 1.030) together with empirical amino acid equilibrium frequencies (F) and the invariant site model (I). The phylogenetic tree was generated in PhyML 3.0 [49] (http://www.atgc-montpellier.fr/phyml/) using this model with Nearest-Neighbor-Interchange (NNI) improvement and Shimodaira-Hasegawa approximate Likelihood Ratio Test (SH-aLRT) branch support. The tree was rooted against *Trichoplax adhaerens* (Fig. 2a) (Additional file 4: Figure S3).

### Co-localization of p53/p63/p73 genes on the same paralogous block

The human p53 gene (ENSG00000141510) is located at chromosome 17: 7,661,779-7,687,550, the p63 gene (ENSG00000073282) is found at chromosome 3: 189,631,416-189,897,279 and the p73 gene (ENSG00000078900) is located at chromosome 1: 3,652,520-3,736,201 (Fig. 3a). Searching the http://wolfe.ucd.ie/dup/human5.28/ homepage [18] a paralogous block in the human genome comprises chromosome 17 (5,01-8,10) and chromosome 3 (167,0-187,25), chromosome 1 (0,76-11,92) and chromosome 3(144,91-185,57), which means that all genes belonging to the p53/p63/p73 family are located on or in close proximity of the same paralogous block (Fig. 3a). VAMP2 (ENSG00000220205) is located in the proximity (5 Mb) of p53, and it has a paralog gene, VAMP3, in the proximity of p73 (10 Mb) which further confirms that these genes are a result from the whole genome duplications. The multicellular organism *Trichoplax adhaerens* contains a single gene of p53/63/73 (TriadG64021) located on scaffold 6 and VAMP2 give a TBLASTN hit on scaffold 6 as well.

### Identification of MDM genes

MDM2 was identified using a TBLASTN search in Ensembl [43] (www.ensembl.org) and its gene tree (ENSGT00530000063539) was downloaded containing 142 MDM2 and MDM4 protein sequences. Additional sequences were collected using TBLASTN human MDM2 (ENST00000258149) as a query. MDM sequences lacking the p53/p63/p73BD were removed. The databases used for browsing and downloading additional sequences were Ensembl Metazoa (www.metazoa.ensembl.org), Pre Ensembl (http://pre.ensembl.org/index.html), NCBI (www.ncbi.nlm.nih.gov), Skatebase [50] (http://skatebase.org), Elephant Shark Genome project [51] (http://esharkgenome.imcb.a-star.edu.sg/), Japanese lamprey genome project (http://jlampreygenome.imcb.a-star.edu.sg/), EchinoBase [52] (www.echinobase.org), MOSAS amphioxus (http://genome.bucm.edu.cn/lancelet/download_data.php), Uniprot (http://www.uniprot.org/), and *Botryllus schlosseri* genome project [53] (http://botryllus.stanford.edu/botryllusgenome/). MDM proteins contain a well-conserved RING domain responsible for binding zinc, this RING domain differ from other RING domains in the binding motif. The common motif of zinc binding is Cys_3_HisCys_4_, while MDM has a unique motif, Cys_2_His_2_Cys_4_ [28]. Presence of the MDM specific motif in the RING domain was a criterion for keeping the sequence in the alignment. The sequences lacking the p53/p63/p73BD were also removed from the final alignment. The alignment resulted in a total number of 166 MDM sequences from 98 species (Additional file 5: Table S2).

### Alignment and phylogenetic tree of MDM

The amino acid alignment was generated in Guidance [46] (http://guidance.tau.ac.il) using MAFFT algorithm with the advanced option max-iterate set to 1000 and pairwise alignment option set to localpair. The alignment was lightly masked [54] (0.050) so that 98,9% of the amino acids remained. Gaps where removed with a gap tolerance of 95% with Gap Strip/Squeeze v2.1.0 (http://www.hiv.lanl.gov/content/sequence/GAPSTREEZE/gap.html) and this alignment was used to generate the phylogenetic tree. The alignment of the p53/p63/p73BD is presented in Additional file 6: Figure S4 and Sequence Logos of this alignment is presented in Additional file 7: Figure S5. The best-fit model, according to Bayesian information criterion [47] (BIC) was calculated using MEGA 6 [48] and resulted in the Jones-Taylor-Thornton substitution (JTT) model with gamma-shaped function (4 categories, fixed alpha to 1.367) (G) together with the invariant site model (I). The phylogenetic tree was calculated using this model in PhyML 3.0 [49] (http://www.atgc-montpellier.fr/phyml/) with Nearest-Neighbor-Interchange (NNI) improvement and Shimodaira-Hasegawa approximate Likelihood Ratio Test (SH-aLRT) branch support. The tree was rooted against *Trichoplax adhaerens* (Fig. 2b)(Additional file 8: Figure S6).

### Co-localization of MDM genes on the same paralogous block

The human MDM2 gene (ENSG00000135679) is located at chromosome 12: 68,808,172-68,850,686 and the MDM4 gene (ENSG00000198625) is located at chromosome 1: 204,516,379-204,558,120 (Fig. 3b). Searching the http://wolfe.ucd.ie/dup/human5.28/ homepage [18] there is a paralogous block located on chromosome 1 (205,69-211,23) and 12 (70,14-98,25), which is in the proximity where MDM2 and MDM4 genes are located (Fig. 3b). Two other genes called PPP1R12A (ENSG00000058272) and MYF5 (ENSG00000111049) are located in the proximity (12 Mb) of MDM2 and have paralog genes, PPP1R12B (ENSG00000077157) and MYOG (ENSG000001221809) in the proximity of MDM4 (3 Mb). Thus, the genes are all located in the proximity of the same paralogous block, which is a result of whole genome duplications (Fig. 3b). The multicellular organism *Trichoplax adhaerens* contains an MDM ancestor (TriadG54791) located on scaffold 3:7,103,976-7,107,199. MYOG and PPP1R12A give a TBLAST hit on scaffold 3 as well, TriadG54311 and TriadG54295 respectively.

## Additional files

Additional file 1: Table S1. Identification list of all p53/p63/p73 sequences that are in the phylogenetic tree in Fig. 2a. The species included are itemized according to phyla and paralog where the Latin name, sequence ID and database is listed. (PDF 81 kb)

Additional file 2: Figure S1. Alignment of the TAD in the p53/p63/p73 protein family. This alignment together with the alignment of the rest of the protein (not shown) was used to generate the phylogenetic tree. The color-coding is according to the eBioX alignment tool. (PDF 106 kb)

Additional file 3: Figure S2. Sequences Logos based on the multiple sequence alignment of p53/p63/p73 TAD. The color-coding is according to the eBioX alignment tool. (PDF 13 kb)

Additional file 4: Figure S3. Phylogenetic tree with support values based on multiple sequence alignment of the p53/p63/p73 protein family only including species with the TAD. Support values are presented as numbers between 0 and 1 in a color gradient between red and blue. (PDF 94 kb)

Additional file 5: Table S2. Identification list of all MDM sequences that are in the phylogenetic tree in Fig. 2B. The species included are itemized according to phyla and paralog where the Latin name, sequence ID and database are listed. (PDF 74 kb)

Additional file 6: Figure S4. Alignment of the p53/p63/p73BD in the MDM protein family. This alignment together with the alignment of the rest of the protein (not shown) was used to generate the phylogenetic tree. The color-coding is according to the eBioX alignment tool. (PDF 3715 kb)

Additional file 7: Figure S5. Sequence Logos based on the multiple sequence alignment of MDM p53/p63/p73BD. The color-coding is according to the eBioX alignment tool. (PDF 25 kb)

Additional file 8: Figure S6. Phylogenetic tree with support values based on multiple sequence alignment of the MDM protein family only including species with the p53/p63/p73 BD. Support values are presented as numbers between 0 and 1 in a color gradient between red and blue. (PDF 21 kb)
