# Supplementary material for: Evolution of the p53-MDM2 pathway
Source: BMC Evol Biol. 2017 Aug 3;17:177. doi: 10.1186/s12862-017-1023-y (PMC5543598; doi:10.1186/s12862-017-1023-y)
Supplement: Supplementary file 1 — Identification list of all p53/p63/p73 sequences that are in the phylogenetic tree in Fig. 2a. The species included are itemized according to phyla and paralog where the Latin name, sequence ID and database is listed. (PDF 81 kb) [file 12862_2017_1023_MOESM1_ESM.pdf]

	Latin name	Sequence ID	Database
**p53/p63/p73**			
**Placozoa**			
	Trichoplax adhaerens	B3RZS6	Uniprot
**Protostomia**			
**Annelida**			
	Capitella teleta	R7UHV7	Uniprot
**Mollusca**			
	Crassostrea gigas	K1RC48	Uniprot
	Euprymna scolopes	Q0H3B6	Uniprot
	Haliotis tuberculata	Q0JRM9	Uniprot
	Loligo forbesii	Q27937	Uniprot
	Lottia gigantea	V4A869	Uniprot
	Mya arenaria	Q9NGC7	Uniprot
	Mytilus edulis	Q1AMZ8	Uniprot
	Mytilus galloprovincialis	R4JHU7	Uniprot
	Mytilus trossulus	Q539B9	Uniprot
	Spisula solidissima	Q6WG19	Uniprot
**Hemichordata**			
	Saccoglossus kowalevskii	XP_002732135 + XP_002738810	NCBI
**Echinodermata**			
	Patiria miniata	Scaffold 74: JH768681 (Genescan)	Echinobase
	Strongylocentrotus purpuratus	H9N2D3	Uniprot
**Deuterostomia**			
	Branchiostoma belcheri	V9I5U2	Uniprot
	Branchiostoma floridae	Brafl1_Scaffold_14_67483	JGI
	Ciona intestinalis	ENSCINP00000003956	Ensembl
	Ciona intestinalis	ENSCINP00000004447	Ensembl
	Ciona savignyi	ENSCSAVP00000016940	Uniprot
	Ciona savignyi	ENSCSAVP00000016659	Ensembl
**Chordata**			
**p53**			
**Agnatha**	Lethenteron camtschaticum	KT960978	NCBI
**Chondrichthyes**			
	Callorhinchus milii	G9J1L8	Uniprot
**Osteichyes**			
	Anabas testudineus	R9XXS5	Uniprot
	Astyanax mexicanus	ENSAMXP00000007265	Ensembl
	Astyanax mexicanus	ENSAMXP00000021366	Ensembl
	Austrofundulus limnaeus	XP_013874967	NCBI
	Barbus barbus	Q9W678	Uniprot
	Clupea harengus	XP_012690246	NCBI
	Coregonus lavaretus	B5TJK8	Uniprot
	Danio rerio	ENSDARP00000051548	Ensembl
	Epinephelus coioides	F8RKR1	Uniprot
	Esox lucius	XM_010867924	NCBI
	Fundulus heteroclitus	Funhe5EG029096t1	OrthoDB7 EOG7JQBNW
	Gasterosteus aculeatus	ENSGACP00000025614	Ensembl
	Haplochromis burtoni	XM_005939679	NCBI
	Ictalurus punctatus	O93379	Uniprot
	Latimeria chalumnae	ENSLACP00000015915	Ensembl
	Lepisosteus oculatus	ENSLOCP00000017063	Ensembl
	Maylandia zebra	XP_004571233	NCBI
	Neolamprologus brichardi	XP_006807759	NCBI
	Nothobranchius furzeri	B3TLB0	Uniprot
	Nothobranchius kuhntae	C6ERD9	Uniprot
	Oncorhynchus keta	Q9W681	Uniprot
	Oncorhynchus kisutch	Q9W680	Uniprot
	Oncorhynchus mykiss	P25035	Uniprot
	Oncorhynchus tshawytscha	Q9W682	Uniprot
	Oreochromis niloticus	ENSONIP00000023874	Ensembl
	Oreochromis niloticus	D5KTJ0	Uniprot
	Oryzias latipes	P79820	Uniprot
	Oryzias latipes	ENSORLP00000008029	Ensembl
	Platichthys flesus	O12946	Uniprot
	Poecilia formosa	ENSPFOP00000007556	Ensembl
	Poecilia formosa	kPFO7556	NCBI
	Poecilia latipinna	XP_014885180	NCBI
	Poecilia mexicana	XP_014844801	NCBI
	Poecilia reticulata	XM_008435413	NCBI
	Salmo salar	C0H8X1	Uniprot
	Salmo salar	C0PUM1	Uniprot

	Siniperca chuatsi	AMR06574	NCBI
	Sinocyclocheilus anshuiensis	XP_016321635	NCBI
	Sinocyclocheilus grahami	XP_016147180	NCBI
	Sinocyclocheilus rhinocerous	XP_016391430	NCBI
	Tachysurus fulvidraco	F5A7P3	Uniprot
	Takifugu rubripes	ENSTRUP00000030645	Ensembl
	Tetraodon miurus	Q9W679	Uniprot
	Tetraodon nigroviridis	ENSTNIP00000013035	Ensembl
	Xiphophorus hellerii	O57538	Uniprot
	Xiphophorus maculatus	ENSXMAP00000015753	Ensembl
**Amphibia**			
	Cynops orientalis	L7NCR5	Uniprot
	Xenopus laevis	P07193	NCBI
	Xenopus tropicalis	ENSXETT00000053759	Ensembl
**Reptilia**			
	Anolis carolinensis	XP_008123458	NCBI
	Chalcides ocellatus	transcript_297464	Reptilian transcriptomes
	Chelonia mydas	XM_007054102	NCBI
	Chrysemys picta bellii	XM_005279339	NCBI
	Crotalus horridus	T1D7I6	Uniprot
	Gekko japonicus	XP_015279412	NCBI
	Pelodiscus sinensis	ENSPSIP00000014905	Ensembl
	Protobothrops mucrosquamatus	XP_015683902	NCBI
	Python bivittatus	XM_007433283	NCBI
	Sphenodon punctatus	25_Locus_3883	Reptilian transcriptomes
	Thamnophis sirtalis	XP_013907681	NCBI
**Marsupialia**			
	Sarcophilus harrisii	ENSSHAP00000018268	Ensembl
**Mammalia**	Ailuropoda melanoleuca	ENSAMEP00000017825	Ensembl
	Bos taurus	ENSBTAP00000001420	Ensembl
	Bubalus bubalis	F6MDM8	Uniprot
	Callithrix jacchus	ENSCJAP00000027663	Ensembl
	Camelus ferus	T0MFN1	Uniprot
	Canis lupus familiaris	ENSCAFP00000024579	Ensembl
	Cavia porcellus	ENSCPOP00000012841	Ensembl
	Chlorocebus sabaeus	ENSCSAP00000007551	Ensembl
	Chlorocebus aethiops	P13481	Uniprot
	Dasypus novemcinctus	ENSDNOP00000004075	Ensembl
	Delphinapterus leucas	Q8SPZ3	Uniprot
	Echinops telfairi	ENSETEP00000007296	Ensembl
	Echinops telfairi	ENSETEP00000008111	Ensembl
	Eospalax baileyi	L7X0Y9	Uniprot
	Eospalax cansus	L7X1P3	Uniprot
	Erinaceus europaeus	ENSEEUP00000013295	Ensembl
	Felis catus	ENSFCAP00000008925	Ensembl
	Gorilla gorilla	ENSGGOP00000009561	Ensembl
	Heterocephalus glaber	G5B5D6	Uniprot
	Homo sapiens	ENSP00000269305	Ensembl
	Ictidomys tridecemlineatus	ENSSTOP00000019678	Ensembl
	Loxodonta africana	ENSLAFP00000027253	Ensembl
	Loxodonta africana	ENSLAFP00000025852	Ensembl
	Loxodonta africana	ENSLAFP00000018379	Ensembl
	Loxodonta africana	ENSLAFP00000027515	Ensembl
	Loxodonta africana	ENSLAFP00000023554	Ensembl
	Loxodonta africana	ENSLAFP00000024998	Ensembl
	Loxodonta africana	ENSLAFP00000023389	Ensembl
	Loxodonta africana	ENSLAFP00000027808	Ensembl
	Loxodonta africana	ENSLAFP00000006292	Ensembl
	Loxodonta africana	ENSLAFP00000028793	Ensembl
	Loxodonta africana	ENSLAFP00000028173	Ensembl
	Macaca mulatta	ENSMMUP00000035274	Ensembl
	Marmota monax	O36006	Uniprot
	Mastomys natalensis	P89002	Uniprot
	Mesocricetus auratus	Q00366	Uniprot
	Microcebus murinus	ENSMICP00000013539	Ensembl
	Microtus oeconomus	L7X447	Uniprot
	Microtus oeconomus	Q920Y0	Uniprot
	Mus musculus	ENSMUSP00000104298	Ensembl
	Mustela putorius furo	ENSMPUP00000008945	Ensembl
	Myotis davidii	L5M0C7	Uniprot
	Myotis lucifugus	ENSMLUP00000005861	Ensembl

	Nannospalax judaei	Q68VB0	Uniprot
	Nomascus leucogenys	ENSNLEP00000011861	Ensembl
	Ochotona princeps	ENSOPRP00000005906	Ensembl
	Odobenus rosmarus divergens	gene13372	OrthoDB7 EOG7JQBNW
	Orcinus orca	gene10761	OrthoDB7 EOG7JQBNW
	Oryctolagus cuniculus	ENSOCUP00000000989	Ensembl
	Oryctolagus cuniculus	ENSOCUP00000025959	Ensembl
	Otolemur garnettii	ENSOGAP00000015106	Ensembl
	Ovis aries	ENSOARP00000016707	Ensembl
	Pan troglodytes	ENSPTRP00000014836	Ensembl
	Papio anubis	ENSPANP00000019643	Ensembl
	Pongo abelii	ENSPPYP00000008923	Ensembl
	Procavia capensis	ENSPCAP00000011892	Ensembl
	Pteropus vampyrus	ENSPVAP00000015400	Ensembl
	Rattus norvegicus	ENSRNOP00000074031	Ensembl
	Sorex araneus	ENSSARP00000005616	Ensembl
	Sus scrofa	ENSSSCP00000019016	Ensembl
	Trichechus manatus	gene15393	OrthoDB7 EOG7JQBNW
	Trichechus manatus	gene25871	OrthoDB7 EOG7JQBNW
	Tupaia belangeri	ENSTBEP00000010189	Ensembl
	Tursiops truncatus	ENSTTRP00000009721	Ensembl
**p63**			
**Chondrichthyes**			
	Callorhinchus milii	G9J1L9	Uniprot
**Osteichyes**			
	Austrofundulus limnaeus	XM_014023050	NCBI
	Cynoglossus semilaevis	XM_008338584	NCBI
	Cyprinodon variegatus	XM_015380973	NCBI
	Fundulus heteroclitus	Funhe5EG030555t1	OrthoDB7 EOG7JQBNW
	Haplochromis burtoni	XM_005919721	NCBI
	Larimichthys crocea	XM_010739540	NCBI
	Latimeria chalumnae	ENSLACT00000025973	Ensembl
	Lepisosteus oculatus	ENSLOCP00000006202	Ensembl
	Maylandia zebra	XM_004552828	NCBI
	Oreochromis niloticus	ENSONIP00000024326	Ensembl
	Oryzias latipes	ENSORLP00000019616	Ensembl
	Poecilia formosa	ENSPFOP00000001124	Ensembl
	Poecilia latipinna	XM_015057236	NCBI
	Poecilia mexicana	XM_014981733	NCBI
	Poecilia reticulata	XM_008407657	NCBI
	Pundamilia nyererei	XM_005727158	NCBI
	Takifugu rubripes	ENSTRUP00000008025	Ensembl
	Tetraodon nigroviridis	ENSTNIP00000016776	Ensembl
	Xiphophorus maculatus	XM_005804434	NCBI
**Aves**			
	Anas platyrhynchos	ENSAPLP00000012746	Ensembl
	Anser cygnoides domesticus	XM_013195004	NCBI
	Aptenodytes forsteri	XM_009282360	NCBI
	Apteryx australis mantelli	XM_013959222	NCBI
	Aquila chrysaetos canadensis	XM_011581144	NCBI
	Calypte anna	XM_008493322	NCBI
	Chaetura pelagica	XM_009999848	NCBI
	Charadrius vociferus	XM_009883358	NCBI
	Chlamydotis macqueenii	XM_010117461	NCBI
	Columba livia	XM_005513215	NCBI
	Corvus brachyrhynchos	XM_008644566	NCBI
	Corvus cornix cornix	XM_010397194	NCBI
	Coturnix japonica	XM_015871911	NCBI
	Cuculus canorus	XM_009565617	NCBI
	Egretta garzetta	XM_009635615	NCBI
	Falco peregrinus	XM_005237743	NCBI
	Ficedula albicollis	ENSFALP00000007718	Ensembl
	Gallus gallus	ENSGALP00000011836	Ensembl
	Geospiza fortis	XM_005418960	NCBI
	Haliaeetus leucocephalus	XM_010573065	NCBI
	Manacus vitellinus	XM_008924515	NCBI
	Meleagris gallopavo	ENSMGAP00000001036	Ensembl
	Melopsittacus undulatus	XM_005142318	NCBI
	Nipponia nippon	XM_009468448	NCBI
	Opisthocomus hoazin	XM_009931665	NCBI
	Parus major	XM_015638327	NCBI
	Picoides pubescens	XM_009901033	NCBI

	Pseudopodoces humilis	XM_005524985	NCBI
	Pygoscelis adeliae	XM_009334097	NCBI
	Serinus canaria	XM_009089139	NCBI
	Struthio camelus australis	XM_009682140	NCBI
	Sturnus vulgaris	XM_014891586	NCBI
	Taeniopygia guttata	ENSTGUP00000009686	Ensembl
	Tinamus guttatus	XM_010222665	NCBI
	Zonotrichia albicollis	XM_005484912	NCBI
**Reptilia**			
	Gekko japonicus	XM_015427170	NCBI
**Marsupialia**			
	Macropus eugenii	ENSMEUP000000014131	Ensembl
	Monodelphis domestica	ENSMODP00000018831	Ensembl
	Ornithorhynchus anatinus	ENSOANP00000021753	Ensembl
	Sarcophilus harrisii	ENSSHAP00000008666	Ensembl
**Mammalia**			
	Ailuropoda melanoleuca	ENSAMEP00000000831	Ensembl
	Bos taurus	ENSBTAP00000020544	Ensembl
	Callithrix jacchus	ENSCJAP00000026376	Ensembl
	Canis lupus familiaris	ENSCAFP00000020585	Ensembl
	Cavia porcellus	ENSCPOP00000013308	Ensembl
	Choloepus hoffmanni	ENSCHOP00000009300	Ensembl
	Dasypus novemcinctus	ENSDNOP00000012853	Ensembl
	Dipodomys ordii	ENSDORP00000001930	Ensembl
	Echinops telfairi	ENSETEP00000001864	Ensembl
	Equus caballus	ENSECAP00000016315	Ensembl
	Felis catus	ENSFCAP00000000731	Ensembl
	Gorilla gorilla	ENSGGOP00000020551	Ensembl
	Heterocephalus glaber	G5CA58	Uniprot
	Homo sapiens	ENSP00000264731	Ensembl
	Loxodonta africana	ENSLAFP00000020806	Ensembl
	Macaca mulatta	ENSMMUP00000021634	Ensembl
	Microcebus murinus	ENSMICP00000004781	Ensembl
	Mus musculus	ENSMUSP00000110965	Ensembl
	Myotis davidii	L5LRF0	Uniprot
	Myotis lucifugus	ENSMLUP00000014213	Ensembl
	Nomascus leucogenys	ENSNLEP00000008197	Ensembl
	Ochotona princeps	ENSOPRP00000002632	Ensembl
	Odobenus rosmarus divergens	gene46131	OrthoDB7 EOG7JQBNW
	Orcinus orca	gene33571	OrthoDB7 EOG7JQBNW
	Ovis aries	ENSOARP00000021997	Ensembl
	Pan troglodytes	ENSPTRP00000027067	Ensembl
	Papio anubis	ENSPANP00000003169	Ensembl
	Pongo abelii	ENSPPYP00000016106	Ensembl
	Pteropus vampyrus	ENSPVAP00000008543	Ensembl
	Rattus norvegicus	ENSRNOP00000033463	Ensembl
	Sorex araneus	ENSSARP00000011553	Ensembl
	Sus scrofa	ENSSSCP00000025960	Ensembl
	Tarsius syrichta	ENSTSYP00000003403	Ensembl
	Trichechus manatus	gene27330	OrthoDB7 EOG7JQBNW
	Tupaia belangeri	ENSTBEP00000003655	Ensembl
	Tursiops truncatus	ENSTTRP00000011034	Ensembl
	Vicugna pacos	ENSVPAP00000011479	Ensembl
**p73**			
**Chondrichthyes**			
	Callorhinchus milii	G9J1M0	Uniprot
**Osteichyes**			
	Astyanax mexicanus	ENSAMXP00000014358	Ensembl
	Austrofundulus limnaeus	XM_014001649	NCBI
	Barbus barbus	Q9W664	Uniprot
	Clupea harengus	XM_012840919	NCBI
	Cynoglossus semilaevis	XM_008317990	NCBI
	Cyprinodon variegatus	XM_015377794	NCBI
	Danio rerio	ENSDARP00000109674	Ensembl
	Esox lucius	XM_010875682	NCBI
	Gadus morhua	ENSGMOP00000016675	Ensembl
	Gasterosteus aculeatus	ENSGACP00000008676	Ensembl
	Haplochromis burtoni	XM_014331015	NCBI
	Larimichthys crocea	XM_010749660	NCBI
	Latimeria chalumnae	ENSLACT00000021843	Ensembl
	Lepisosteus oculatus	ENSLOCP00000006343	Ensembl
	Maylandia zebra	XM_004554110	NCBI

	Neolamprologus brichardi	XM_006785472	NCBI
	Notothenia coriiceps	XM_010774882	NCBI
	Oreochromis niloticus	ENSONIP00000002627	Ensembl
	Oreochromis niloticus	XM_003444786	NCBI
	Poecilia formosa	ENSPFOP00000010896	Ensembl
	Poecilia latipinna	XM_015015929	NCBI
	Poecilia mexicana	XM_014973019	NCBI
	Poecilia reticulata	XM_008414745	NCBI
	Pundamilia nyererei	XM_005732992	NCBI
	Salmo salar	XM_014146164	NCBI
	Sinocyclocheilus grahami	XM_016294985	NCBI
	Stegastes partitus	XM_008288970	NCBI
	Takifugu rubripes	ENSTRUP00000038165	Ensembl
	Tetraodon nigroviridis	ENSTNIP00000014938	Ensembl
**Amphibia**			
	Xenopus tropicalis	ENSXETT00000010981	Ensembl
**Reptilia**			
	Anolis carolinensis	ENSACAP00000001746	Ensembl
	Pelodiscus sinensis	ENSPSIP00000004448	Ensembl
**Aves**			
	Anas platyrhynchos	ENSAPLP00000003415	Ensembl
	Anser cygnoides domesticus	XM_013194785	NCBI
	Aptenodytes forsteri	XM_009274116	NCBI
	Calypte anna	XM_008503635	NCBI
	Caprimulgus carolinensis	XM_010165570	NCBI
	Cariama cristata	XM_009704081	NCBI
	Chaetura pelagica	XM_010005289	NCBI
	Charadrius vociferus	XM_009889627	NCBI
	Columba livia	XM_005513215	NCBI
	Corvus brachyrhynchos	XM_008641249	NCBI
	Corvus cornix cornix	XM_010405845	NCBI
	Coturnix japonica	XM_015882545	NCBI
	Cuculus canorus	XM_009561561	NCBI
	Ficedula albicollis	ENSFALP00000010031	Ensembl
	Gallus gallus	XM_015297092	NCBI
	Geospiza fortis	XM_005427649	NCBI
	Manacus vitellinus	XM_008928758	NCBI
	Meleagris gallopavo	XM_010722720	NCBI
	Melopsittacus undulatus	XM_013127520	NCBI
	Nipponia nippon	XM_009460860	NCBI
	Parus major	XM_015648140	NCBI
	Pseudopodoces humilis	XM_014257222	NCBI
	Pygoscelis adeliae	XM_009320554	NCBI
	Pygoscelis adeliae	XM_009320547	NCBI
	Serinus canaria	XM_009095577	NCBI
	Struthio camelus australis	XM_009671626	NCBI
	Taeniopygia guttata	ENSTGUP00000002829	Ensembl
	Tinamus guttatus	XM_010224396	NCBI
	Zonotrichia albicollis	XM_014271078	NCBI
**Marsupialia**			
	Macropus eugenii	ENSMEUP00000000705	Ensembl
**Mammalia**			
	Ailuropoda melanoleuca	ENSAMEP00000009745	Ensembl
	Bos taurus	ENSBTAP00000007643	Ensembl
	Camelus ferus	S9YSN3	Uniprot
	Canis lupus familiaris	ENSCAFP00000028819	Ensembl
	Cavia porcellus	ENSCPOP00000009800	Ensembl
	Chlorocebus aethiops	Q9XSK8	Uniprot
	Chlorocebus sabaeus	ENSCSAP00000017283	Ensembl
	Dasypus novemcinctus	ENSDNOP00000028722	Ensembl
	Dipodomys ordii	ENSDORP00000013966	Ensembl
	Echinops telfairi	ENSETEP00000007165	Ensembl
	Erinaceus europaeus	ENSEEUP00000011975	Ensembl
	Felis catus	ENSFCAP00000010792	Ensembl
	Gorilla gorilla	ENSGGOP00000015180	Ensembl
	Heterocephalus glaber	G5CBI6	Uniprot
	Homo sapiens	ENSP00000367545	Ensembl
	Macaca fascicularis	G7NTA7	Uniprot
	Macaca mulatta	ENSMMUP00000021768	Ensembl
	Mus musculus	ENSMUSP00000101269	Ensembl
	Mustela putorius furo	G9KUR7	Uniprot
	Nomascus leucogenys	ENSNLEP00000009887	Ensembl

	Odobenus rosmarus divergens	gene32427	OrthoDB7 EOG7JQBNW
	Orcinus orca	gene21122	OrthoDB7 EOG7JQBNW
	Otolemur garnettii	ENSOGAP00000005402	Ensembl
	Ovis aries	ENSOARP00000017005	Ensembl
	Ovis aries	W5Q2R4	Uniprot
	Pan troglodytes	ENSPTRP00000000118	Ensembl
	Papio anubis	ENSPANP00000007545	Ensembl
	Pongo abelii	ENSPPYP00000002277	Ensembl
	Procavia capensis	ENSPCAP00000000662	Ensembl
	Pteropus vampyrus	ENSPVAP00000008433	Ensembl
	Rattus norvegicus	ENSRNOP00000052265	Ensembl
	Sus scrofa	ENSSSCP00000022771	Ensembl
	Trichechus manatus latirostris	gene31754	OrthoDB7 EOG7JQBNW
	Tursiops truncatus	ENSTTRP00000003223	Ensembl
