# Supplementary figures and images for: Evolution of the p53-MDM2 pathway
Source: BMC Evol Biol. 2017 Aug 3;17:177. doi: 10.1186/s12862-017-1023-y (PMC5543598; doi:10.1186/s12862-017-1023-y)

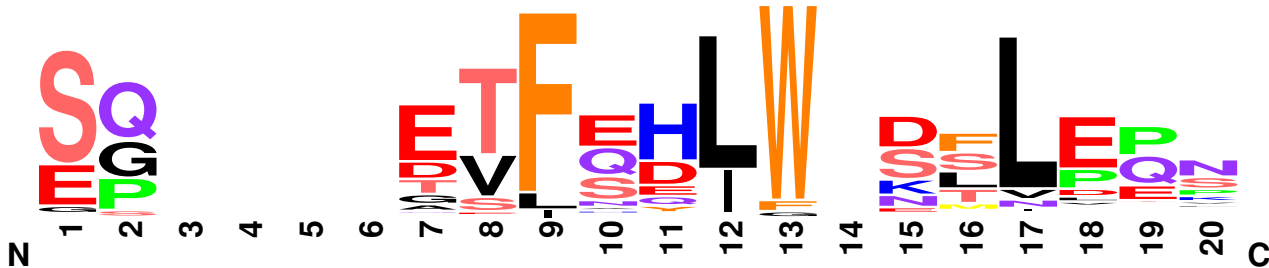

Amino acid position

Supplement: Supplementary file 3 — Sequences Logos based on the multiple sequence alignment of p53/p63/p73 TAD. The color-coding is according to the eBioX alignment tool. (PDF 13 kb) [file 12862_2017_1023_MOESM3_ESM.pdf]

Tree scale: 1

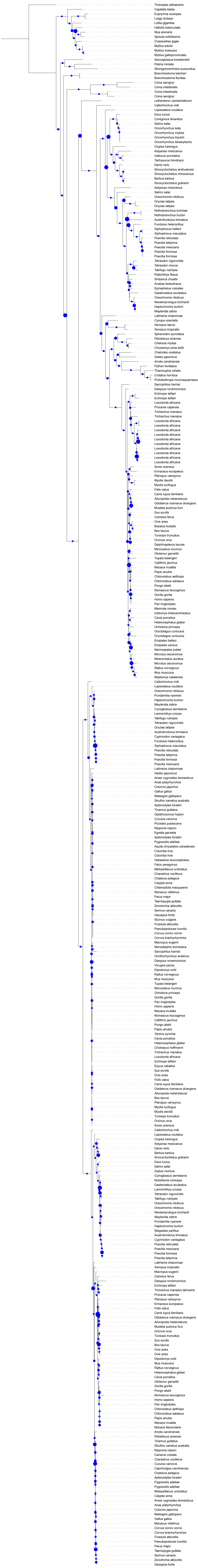

Supplement: Supplementary file 4 — Phylogenetic tree with support values based on multiple sequence alignment of the p53/p63/p73 protein family only including species with the TAD. Support values are presented as numbers between 0 and 1 in a color gradient between red and blue. (PDF 94 kb) [file 12862_2017_1023_MOESM4_ESM.pdf]

N

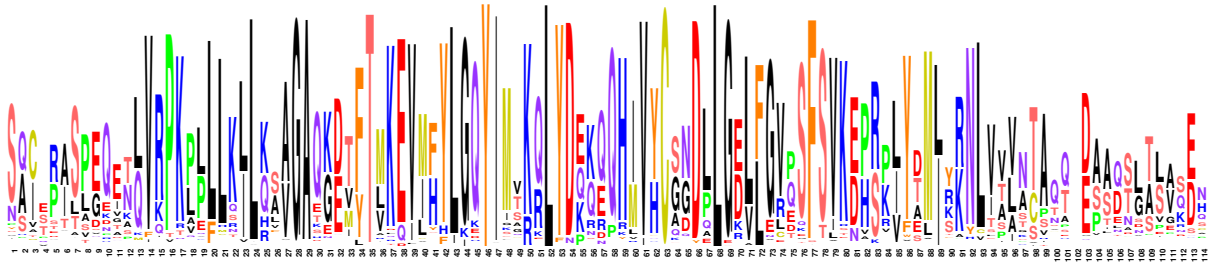

C

Amino acid position

Supplement: Supplementary file 7 — Sequence Logos based on the multiple sequence alignment of MDM p53/p63/p73BD. The color-coding is according to the eBioX alignment tool. (PDF 25 kb) [file 12862_2017_1023_MOESM7_ESM.pdf]

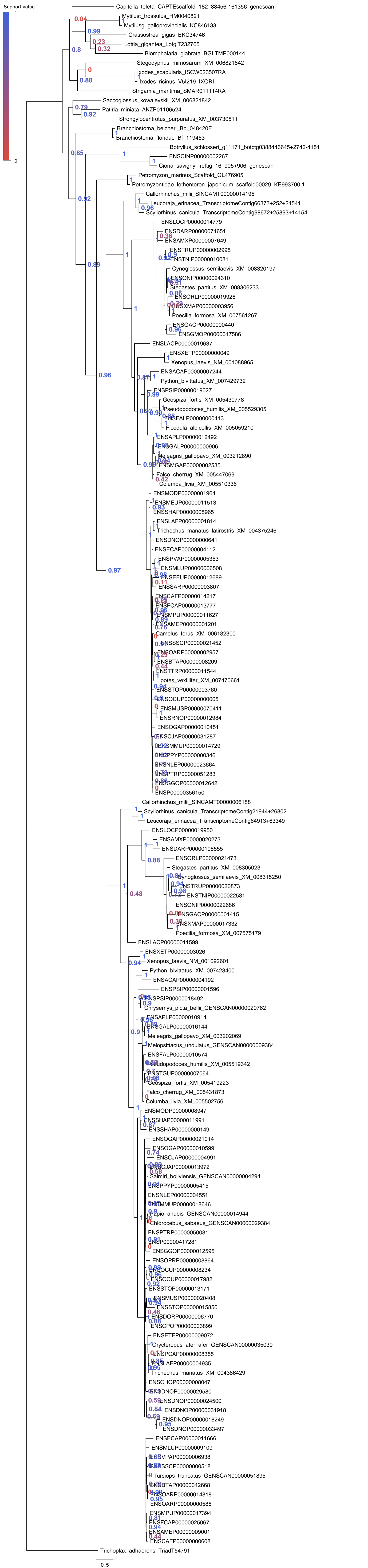

Supplement: Supplementary file 8 — Phylogenetic tree with support values based on multiple sequence alignment of the MDM protein family only including species with the p53/p63/p73 BD. Support values are presented as numbers between 0 and 1 in a color gradient between red and blue. (PDF 21 kb) [file 12862_2017_1023_MOESM8_ESM.pdf]
