# Supplementary material for: Evolution of the p53-MDM2 pathway
Source: BMC Evol Biol. 2017 Aug 3;17:177. doi: 10.1186/s12862-017-1023-y (PMC5543598; doi:10.1186/s12862-017-1023-y)
Supplement: Supplementary file 5 — Identification list of all MDM sequences that are in the phylogenetic tree in Fig. 2B. The species included are itemized according to phyla and paralog where the Latin name, sequence ID and database are listed. (PDF 74 kb) [file 12862_2017_1023_MOESM5_ESM.pdf]

	Latin name	Sequence ID	Database
**MDM**			
Placozoa			
	Trichoplax adhaerens	TriadT54791	Ensembl metazoa
Annelida			
	Capitella teleta	CAPTE Scaffold 18: 88,456-161,356 (Genescan)	Ensembl metazoa
Mollusca			
	Biomphalaria glabrata	BGLTMP000144	VectorBase
	Crassostrea gigas	EKC34746	Ensembl metazoa
	Lottia gigantea	LotgiT232765	Ensembl metazoa
	Mytilusg galloprovincialis	KC846133	NCBI
	Mytilust trossulus	HM0040821	NCBI
Arthropoda: Myriapoda			
	Strigamia maritima	SMAR011114RA	Ensembl metazoa
Arthropoda:Chelicerata			
	Ixodes ricinus	V5I219	Uniprot
	Ixodes scapularis	ISCW023507RA	Ensembl metazoa
	Stegodyphus mimosarum	A0A087UQB2	Uniprot
Hemichordata			
	Saccoglossus kowalevskii	XM_006821842	NCBI
Echinodermata			
	Patiria miniata	AKZP01106524	NCBI
	Strongylocentrotus purpuratus	XM_003730511	NCBI
Deuterostome			
	Botryllus schlosseri	g11171_botctg038844:6645:+:2742-4151	Botryllus Schlosseri Genome Project
	Branchiostoma belcheri	Bb_048420F	LanceletDB
	Branchiostoma floridae	Bf_119453	LanceletDB
	Ciona intestinalis	CINP00000002267	Ensembl
	Ciona savignyi	reftig_16 (Genescan)	Ensembl
Chordata			
	Petromyzontidae lethenteron japonicum	APJL01000000 Scaffold 00029 : KE993700.1 (Genescan)	NCBI
	Petromyzon marinus	Scaffold GL476905 (Genescan)	Ensembl
**MDM2**			
Chondrichthyes			
	Callorhinchus milii	SINCAMT00000006188	Elephant Shark Genome Project
	Leucoraja erinacea	Transcriptome Contig 64913 + 63349	SkateBase
	Scyliorhinus canicula	Transcriptome Contig 21944 + 26802	SkateBase
Osteichyes			
	Astyanax mexicanus	AMXP00000020273	Ensembl
	Cynoglossus semilaevis	XM_008315250	NCBI
	Danio rerio	DARP00000108555	Ensembl
	Gasterosteus aculeatus	GACP00000001415	Ensembl
	Latimeria chalumnae	LACP00000011599	Ensembl
	Lepisosteus oculatus	LOCP00000019950	Ensembl
	Oreochromis nilotics	ONIP00000022686	Ensembl
	Oryzias latipes	ORLP00000021473	Ensembl
	Poecilia formosa	XM_007575179	NCBI
	Stegastes partitus	XM_008305023	NCBI
	Takifugu rubripes	TRUP00000020873	Ensembl
	Tetraodon nigroviridis	TNIP00000022581	Ensembl
	Xiphophorus maculatus	XMAP00000017332	Ensembl
Amphibia			
	Xenopus laevis	NM_001092601	NCBI
	Xenopus tropicalis	XETP00000003026	Ensembl
Reptilia			
	Anolis carolinensis	ACAP00000004192	Ensembl
	Chrysemys picta bellii	GENSCAN00000020762	Pre-ensembl
	Pelodiscus sinensis	PSIP00000018492	Ensembl
	Pelodiscus sinensis	PSIP00000001596	Ensembl
	Python bivittatus	XM_007423400	NCBI
Aves			
	Anas platyrhynchos	APLP00000010914	Ensembl
	Columba livia	XM_005502756	NCBI
	Falco cherrug	XM_005431873	NCBI
	Ficedula albicollis	FALP00000010574	Ensembl
	Gallus gallus	GALP00000016144	Ensembl
	Geospiza fortis	XM_005419223	NCBI
	Meleagris gallopavo	XM_003202069	NCBI
	Melopsittacus undulatus	GENSCAN00000009384	Pre-ensembl
	Pseudopodoces humilis	XM_005519342	NCBI
	Taeniopygia guttata	TGUP00000007064	Ensembl
Marsupials			
	Monodelphis domestica	MODP00000008947	Ensembl
	Sarcophilus harrisii	SHAP00000011991	Ensembl
	Sarcophilus harrisii	SHAP00000000149	Ensembl
Mammals			
	Ailuropoda melanoleuca	AMEP00000009001	Ensembl
	Bos taurus	BTAP00000042668	Ensembl

	Callithrix jacchus	CJAP00000013972	Ensembl
	Callithrix jacchus	CJAP00000004991	Ensembl
	Canis lupus familiaris	CAFP00000000608	Ensembl
	Cavia porcellus	OCUP00000008234	Ensembl
	Cavia porcellus	OCUP00000017982	Ensembl
	Chlorocebus sabaeus	GENSCAN00000029384	Pre-ensembl
	Choleopus hoffmanni	CHOP00000008047	Ensembl
	Dasypus novemcinctus	DNOP00000029580	Ensembl
	Dasypus novemcinctus	DNOP00000031918	Ensembl
	Dasypus novemcinctus	DNOP00000018249	Ensembl
	Dasypus novemcinctus	DNOP00000033497	Ensembl
	Dasypus novemcinctus	DNOP00000024500	Ensembl
	Dipodomys ordii	DORP00000006770	Ensembl
	Echinops telfairi	ETEP00000009072	Ensembl
	Equus caballus	ECAP00000011666	Ensembl
	Felix catus	FCAP00000025067	Ensembl
	Gorilla gorilla	GGOP00000012595	Ensembl
	Homo sapiens	P00000417281	Ensembl
	Ictidomys tridecemlineatus	STOP00000015850	Ensembl
	Ictidomys tridecemlineatus	STOP00000013171	Ensembl
	Loxodonta africana	LAFP00000004935	Ensembl
	Macaca mulatta	MMUP00000018646	Ensembl
	Mus musculus	MUSP00000020408	Ensembl
	Mustela putorius furo	MPUP00000017394	Ensembl
	Myotis lucifugus	MLUP00000009109	Ensembl
	Nomascus leucogenys	NLEP00000004551	Ensembl
	Ochotona princeps	OPRP00000008864	Ensembl
	Orycteropus afer afer	GENSCAN00000035039	Pre-ensembl
	Oryctoiagus cuniculus	CPOP00000003899	Ensembl
	Otolemur garnettii	OGAP00000010599	Ensembl
	Otolemur garnettii	OGAP00000021014	Ensembl
	Ovis aries	OARP000000148181	Ensembl
	Ovis aries	OARP00000000585	Ensembl
	Pan troglodytes	PTRP00000050081	Ensembl
	Papio anubis	GENSCAN00000014944	Pre-ensembl
	Pongo abelii	PPYP00000005415	Ensembl
	Procavia capensis	PCAP00000008355	Ensembl
	Saimiri boliviensis	GENSCAN00000004294	Pre-ensembl
	Sus scrofa	SSCP00000000518	Ensembl
	Trichechus manatus	XM_004386429	NCBI
	Tursiops truncatus	GENSCAN00000051895	Pre-ensembl
	Vicugna pacos	VPAP00000006938	Ensembl
**MDM4**			
Chondrichthyes			
	Callorhinchus milii	SINCAMT00000014195	Elephant Shark Genome Project
	Leucoraja erinacea	Transcriptome Contig 66373 + 252 + 24541	SkateBase
	Scyliorhinus canicula	Transcriptome Contig 98672 + 25893 + 14154	SkateBase
Osteichyes			
	Astyanax mexicanus	AMXP00000007649	Ensembl
	Cynoglossus semilaevis	XM_008320197	NCBI
	Danio rerio	DARP00000074651	Ensembl
	Gadus morhua	GMOP00000017586	Ensembl
	Gasterosteus aculeatus	GACP00000000440	Ensembl
	Latimeria chalumnae	LACP00000019637	Ensembl
	Lepisosteus oculatus	LOCP00000014779	Ensembl
	Oreochromis nilotics	ONIP00000024310	Ensembl
	Oryzias latipes	ORLP00000019926	Ensembl
	Poecilia formosa	XM_007561267	NCBI
	Stegastes partitus	XM_008306233	NCBI
	Takifugu rubripes	TRUP00000002995	Ensembl
	Tetraodon nigroviridis	TNIP00000010081	Ensembl
	Xiphophorus maculatus	XMAP00000003956	Ensembl
Amphibia			
	Xenopus laevis	NM_001088965	NCBI
	Xenopus tropicalis	XETP00000000049	Ensembl
Reptilia			
	Anolis carolinensis	ACAP00000007244	Ensembl
	Pelodiscus sinensis	PSIP00000019027	Ensembl
	Python bivittatus	XM_007429732	NCBI
Aves			
	Anas platyrhynchos	APLP00000012492	Ensembl
	Columba livia	XM_005510336	NCBI
	Falco cherrug	XM_005447069	NCBI
	Ficedula albicollis	FALP00000000413	Ensembl
	Ficedula albicollis	XM_005059210	NCBI
	Gallus gallus	GALP00000000906	Ensembl
	Geospiza fortis	XM_005430778	NCBI
	Meleagris gallopavo	MGAP00000002535	Ensembl

	Meleagris gallopavo	XM_003212890	NCBI
	Pseudopodoces humilis	XM_005529305	NCBI
Marsupials			
	Macropus eugenii	MEUP00000011513	Ensembl
	Monodelphis domestica	MODP00000001964	Ensembl
	Sarcophilus harrisii	SHAP00000008965	Ensembl
Mammals			
	Ailuropoda melanoleuca	AMEP00000001201	Ensembl
	Bos taurus	BTAP00000008209	Ensembl
	Callithrix jacchus	CJAP00000031287	Ensembl
	Camelus ferus	XM_006182300	NCBI
	Canis lupus familiaris	CAFP00000014217	Ensembl
	Cavia porcellus	OCUP00000000005	Ensembl
	Dasypus novemcinctus	DNOP00000000641	Ensembl
	Equus caballus	ECAP00000004112	Ensembl
	Erinaceus europaeus	EEUP00000012689	Ensembl
	Felix catus	FCAP00000013777	Ensembl
	Gorilla gorilla	GGOP00000012642	Ensembl
	Homo sapiens	P00000356150	Ensembl
	Ictidomys tridecemlineatus	STOP00000003760	Ensembl
	Lipotes vexillifer	XM_007470661	NCBI
	Loxodonta africana	LAFP00000001814	Ensembl
	Macaca mulatta	MMUP00000014729	Ensembl
	Mus musculus	MUSP00000070411	Ensembl
	Mustela putorius furo	MPUP00000011627	Ensembl
	Myotis lucifugus	MLUP00000006508	Ensembl
	Nomascus leucogenys	NLEP00000023664	Ensembl
	Otolemur garnettii	OGAP00000010451	Ensembl
	Ovis aries	OARP00000002957	Ensembl
	Pan troglodytes	PTRP00000051283	Ensembl
	Pongo abelii	PPYP00000000346	Ensembl
	Pteropus vampyrus	PVAP00000005353	Ensembl
	Rattus norvegicus	RNOP00000012984	Ensembl
	Sorex araneus	SARP00000003807	Ensembl
	Sus scrofa	SSCP00000021452	Ensembl
	Trichechus manatus latirostris	XM_004375246	NCBI
	Tursiops truncates	TTRP00000011544	Ensembl
