# Supplementary material for: Evolution of the p53-MDM2 pathway
Source: BMC Evol Biol. 2017 Aug 3;17:177. doi: 10.1186/s12862-017-1023-y (PMC5543598; doi:10.1186/s12862-017-1023-y)
Supplement: Supplementary file 6 — Alignment of the p53/p63/p73BD in the MDM protein family. This alignment together with the alignment of the rest of the protein (not shown) was used to generate the phylogenetic tree. The color-coding is according to the eBioX alignment tool. (PDF 3715 kb) [file 12862_2017_1023_MOESM6_ESM.pdf]

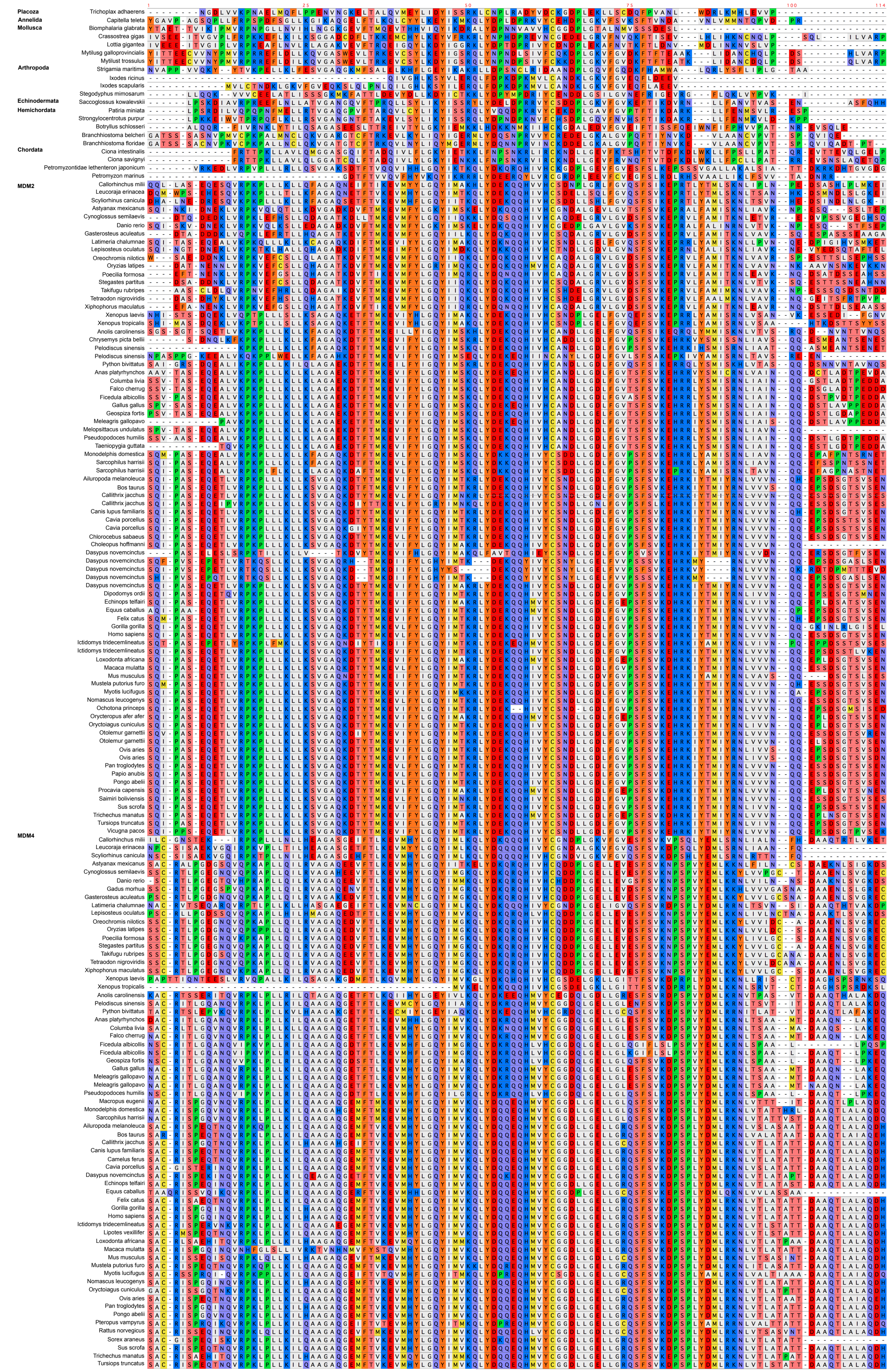

1
25
50
75
100
114
Placoza
Annelida
Mollusca
Arthropoda
Echinodermata
Hemichordata
Chordata
MDM2
MDM4
Trichoplax adhaerens
Capitella teleta
Biomphalaria glabrata
Crassostrea gigas
Lottia gigantea
Mytilusg galloprovincialis
Mytilust trossulus
Strigamia maritima
Ixodes ricinus
Ixodes scapularis
Stegodyphus mimosarum
Saccoglossus kowalevskii
Patiria miniata
Strongylocentrotus purpur
Botryllus schlosseri
Branchiostoma belcheri
Branchiostoma floridae
Ciona intestinalis
Ciona savignyi
Petromyzontidae lethenteron japonicum
Petromyzon marinus
Callorhinchus milii
Leucoraja erinacea
Scyliorhinus canicula
Astyanax mexicanus
Cynoglossus semilaevis
Danio rerio
Gasterosteus aculeatus
Latimeria chalumnae
Lepisosteus oculatus
Oreochromis nilotics
Oryzias latipes
Poecilia formosa
Stegastes partitus
Takifugu rubripes
Tetraodon nigroviridis
Xiphophorus maculatus
Xenopus laevis
Xenopus tropicalis
Anolis carolinensis
Chrysemys picta bellii
Pelodiscus sinensis
Pelodiscus sinensis
Python bivittatus
Anas platyrhynchos
Columba livia
Falco cherrug
Ficedula albicollis
Gallus gallus
Geospiza fortis
Meleagris gallopavo
Melopsittacus undulatus
Pseudopodoces humilis
Taeniopygia guttata
Monodelphis domestica
Sarcophilus harrisii
Sarcophilus harrisii
Ailuropoda melanoleuca
Bos taurus
Callithrix jacchus
Callithrix jacchus
Canis lupus familiaris
Cavia porcellus
Cavia porcellus
Chlorocebus sabaeus
Choleopus hoffmanni
Dasypus novemcinctus
Dasypus novemcinctus
Dasypus novemcinctus
Dasypus novemcinctus
Dasypus novemcinctus
Dipodomys ordii
Echinops telfairi
Equus caballus
Felix catus
Gorilla gorilla
Homo sapiens
Ictidomys tridecemlineatus
Ictidomys tridecemlineatus
Loxodonta africana
Macaca mulatta
Mus musculus
Mustela putorius furo
Myotis lucifugus
Nomascus leucogenys
Ochotona princeps
Orycteropus afer afer
Oryctolagus cuniculus
Otolemur garnettii
Otolemur garnettii
Ovis aries
Ovis aries
Pan troglodytes
Papio anubis
Pongo abelii
Procavia capensis
Saimiri boliviensis
Sus scrofa
Trichechus manatus
Tursiops truncatus
Vicugna pacos
Callorhinchus milii
Leucoraja erinacea
Scyliorhinus canicula
Astyanax mexicanus
Cynoglossus semilaevis
Danio rerio
Gadus morhua
Gasterosteus aculeatus
Latimeria chalumnae
Lepisosteus oculatus
Oreochromis nilotics
Oryzias latipes
Poecilia formosa
Stegastes partitus
Takifugu rubripes
Tetraodon nigroviridis
Xiphophorus maculatus
Xenopus laevis
Xenopus tropicalis
Anolis carolinensis
Pelodiscus sinensis
Python bivittatus
Anas platyrhynchos
Columba livia
Falco cherrug
Ficedula albicollis
Ficedula albicollis
Geospiza fortis
Gallus gallus
Meleagris gallopavo
Meleagris gallopavo
Pseudopodoces humilis
Macropus eugenii
Monodelphis domestica
Sarcophilus harrisii
Ailuropoda melanoleuca
Bos taurus
Callithrix jacchus
Canis lupus familiaris
Camelus ferus
Cavia porcellus
Dasypus novemcinctus
Echinops telfairi
Equus caballus
Felix catus
Gorilla gorilla
Homo sapiens
Ictidomys tridecemlineatus
Lipotes vexillifer
Loxodonta africana
Macaca mulatta
Mus musculus
Mustela putorius furo
Myotis lucifugus
Nomascus leucogenys
Oryctolagus cuniculus
Ovis aries
Pan troglodytes
Pongo abelii
Pteropus vampyrus
Rattus norvegicus
Sorex araneus
Sus scrofa
Trichechus manatus
Tursiops truncatus
