# Supplementary material for: Evolution of the p53-MDM2 pathway
Source: BMC Evol Biol. 2017 Aug 3;17:177. doi: 10.1186/s12862-017-1023-y (PMC5543598; doi:10.1186/s12862-017-1023-y)
Supplement: Supplementary file 2 — Alignment of the TAD in the p53/p63/p73 protein family. This alignment together with the alignment of the rest of the protein (not shown) was used to generate the phylogenetic tree. The color-coding is according to the eBioX alignment tool. (PDF 106 kb) [file 12862_2017_1023_MOESM2_ESM.pdf]

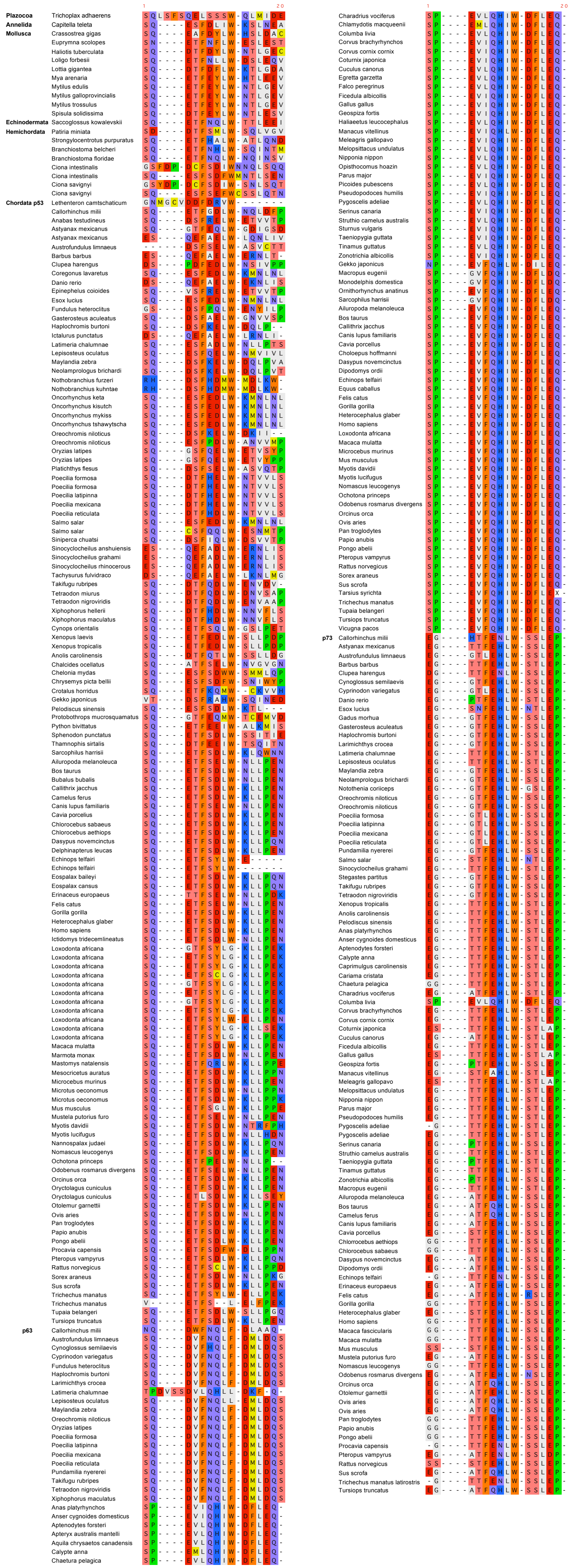

1
20
Plazocoa
Annelida
Mollusca
Echinodermata
Hemichordata
Chordata p53
p63
p73
